# Promoter DNA methylation analysis reveals a combined diagnosis of CpG-based biomarker for prostate cancer

**DOI:** 10.18632/oncotarget.16437

**Published:** 2017-03-22

**Authors:** Yuanyuan Tang, Shusuan Jiang, Yinmin Gu, Weidong Li, Zengnan Mo, Yuanjie Huang, Tianyu Li, Yanling Hu

**Affiliations:** ^1^ Guangxi Reproductive Medical Research Center, First Affiliated Hospital of Guangxi Medical University, Nanning, Guangxi 530021, China; ^2^ Department of Urology, First Affiliated Hospital of Guangxi Medical University, Nanning, Guangxi 530021, China; ^3^ Life Sciences Institute, Guangxi Medical University, Nanning, Guangxi 530021, China; ^4^ Center for Genomic and Personalized Medicine, Guangxi Medical University, Nanning, Guangxi 530021, China; ^5^ Guangxi Colleges and Universities Key Laboratory of Biological Molecular Medicine Research, Guangxi Medical University, Nanning, Guangxi 530021, China

**Keywords:** prostate cancer, DNA methylation, promoter, diagnostic biomarker, gene

## Abstract

**Background:**

Prostate cancer (PCa) is the most common tumor in elderly men. However, the specificity and sensitivity of serum prostate-specific antigen levels in PCa diagnosis are controversial. This study aims to reveal a novel diagnosis biomarker in PCa.

**Materials and Methods:**

The differential methylated CpG sites between 423 primary PCa and 39 adjacent samples from The Cancer Genome Atlas (TCGA) on Illumina HumanMethylation 450 platform were analyzed. The diagnostic methylation markers were mined using the Prediction Analysis of Microarrays package in Bioconductor. Then, the Gene Expression Omnibus data was used for verification. Pyrosequencing was applied to improve methylation levels of five CpGs (cg06363129, cg08843517, cg05385513, cg07220448 and cg11417025).

**Results:**

The area under curve of receiver operating characteristic of eight diagnostic methylation CpGs (cg06363129, cg08843517, cg03576469, cg05385513, cg07220448, cg11417025, cg20883831, and cg23824801) in TCGA data ranged from 0.910 to 0.939. Except for cg20883831 and cg23824801, the correlations between methylation levels of six other sites and their expressions in patients were significant (r > 0.5 and P < 0.001). The methylation level of cg06363129 was significantly different between the groups of Gleason Score (GS) = 7 and GS ≥ 8 (P < 0.05). Pyrosequencing in our samples confirmed that four diagnostic methylation sites (cg06363129, cg08843517, cg05385513, and cg11417025) had high diagnostic efficacy.

**Conclusions:**

The combined diagnosis of four methylation CpGs sites (cg06363129, cg08843517, cg05385513, and cg11417025) in the gene promoter has high tissue specificity and diagnostic efficacy for PCa. Results revealed a novel potential biomarker for prostate cancer diagnosis.

## INTRODUCTION

Prostate cancer (PCa) is the most common cancer in the male reproductive system and is the leading cause of cancer-related mortality in males worldwide [1]. The incidence of PCa is lower in Asian countries compared with European countries. However, given the changes in lifestyle and the declining proportion of the aging population in China, PCa increases faster than other malignant tumors [2]. Currently, prostate -specific antigen (PSA) is widely used as a diagnostic PCa molecular markers in clinical practice. However, due to the impact of benign prostatic hyperplasia, inflammation, age, drug, and other related factors, the specificity and sensitivity of PSA remains controversial [3, 4]. An accurate marker for PCa diagnosis may help doctors and patients implement treatment. Many researchers have been looking for other highly specific diagnosis marker for PCa.

Epigenetic mechanisms that may be involved in the development of PCa have attracted considerable research interest [5, 6]. Epigenetics refers to a DNA sequence that does not change. However, the gene undergoes a heritable change that alters the cell genetic material, and this change can be steadily transferred to cell proliferation. The epigenetic molecular mechanisms include DNA methylation, RNA interference, histone modifications, and chromatin modifications [7], among which DNA methylation is the most common. Previous studies have shown that aberrant DNA methylation is closely related to the development of tumors, such as that in PCa [8], bladder cancer [9], hepatocellular carcinoma [10], nasopharyngeal carcinoma [11], lung neoplasms [12], and gynecologic oncology [13]. Abnormal DNA methylation may occur from gene to site. A variety of cancer-related genes can be expressed in gene promoter methylation, activating the proliferation of tumor cell [5]. However, the diagnosis of PCa using CpG-based biomarkers has been rarely reported.

To explore the relationships between the methylation levels of CpG sites and PCa, we analyzed the differentially methylated sites of 423 primary PCa and 39 adjacent samples from The Cancer Genome Atlas (TCGA) database in this study. First, we utilized bioinformatics methods to mine eight diagnostic methylation sites (cg06363129, cg07220448, and cg11417025 in SOSTDC1; cg08843517 in CYBA; cg03576469 in CCDC8; cg05385513 in EFEMP1; cg20883831in KCNH2; and cg23824801in CBX5) from TCGA. These sites are tissue specific and have high diagnostic efficacy that could distinguish PCa from adjacent samples. The result was further verified using the Gene Expression Omnibus (GEO) dataset. Finally, we tested five methylated sites in the gene promoter (cg06363129, cg08843517, cg05385513, cg07220448, and cg11417025) through pyrosequencing in our samples. The combined diagnosis of four diagnostic methylation sites (cg06363129, cg08843517, cg05385513, and cg11417025) showed high diagnostic efficacy and could be used in clinical research. These results may provide a novel potential biomarker for diagnosis of PCa.

## RESULTS

### Differentially methylated CpG sites between PCa and adjacent samples from TCGA

**Figure 1:**
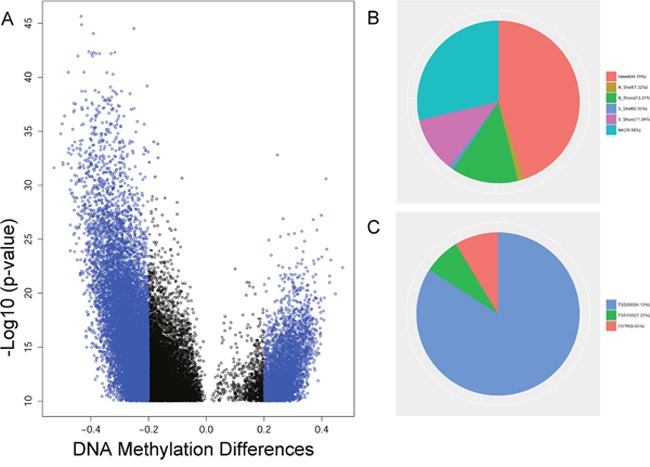
The analysis of differentially methylated sites from TCGA **(A)** The volcano plot of differentially methylated sites with FDR < 1E-10 between the tumor and the adjacent in prostate cancer. **(B)** The distribution of differentially methylated sites in promoter region. **(C)** The distribution of differentially methylated sites in CpG island.

To explore the DNA methylome in PCa, we collected 423 primary PCa and 39 adjacent samples that were profiled through Illumina HumanMethylation450 platform from the TCGA (Table 1). The Illumina HumanMethylation450 array included 482,421 CpG sites. After quality filtering, 445,802 detected sites were analyzed. We used the limma software package for linear regression model analysis. Finally, a total of 15,744 differentially methylated CpGs (False Discovery Rate < 1E-10, |Delta Beta| >0.2) were found between PCa and adjacent samples. After removing the CpGs located in microRNA, 1,585 CpGs in the gene promoters were assayed in this study (Supplementary Table 2). Among these sites, we found that 139 hypermethylated and 1,446 hypomethylated CpGs were present in PCa (Figure 1A). Additionally, we observed that the differentially methylated sites were mainly located in CpG islands (44.79%) and TSS200 region (84.13%) in the gene promoter (Figure 1B and 1C).

**Table 1:** Clinical characteristics of prostate cancer patients from TCGA

Variables	Prostate cancer (n=423)	Adjacent (n=39)
Age(years)	61(56,66)	61(55,66)
**Race**		
White	136	38
Black or African American	7	1
Asian	2	0
NA	278	0
**Tumor size**		
T1	0	0
T2	183	18
T3	230	18
T4	8	3
NA	2	0
**Lymph node**		
N0	299	31
N1	57	2
NA	67	6
**Metastasis status**		
M0	0	0
M1	0	0
NA	423	39

NA: not available. The ages were presented as the median (25%–75% quartiles).

**Figure 2:**
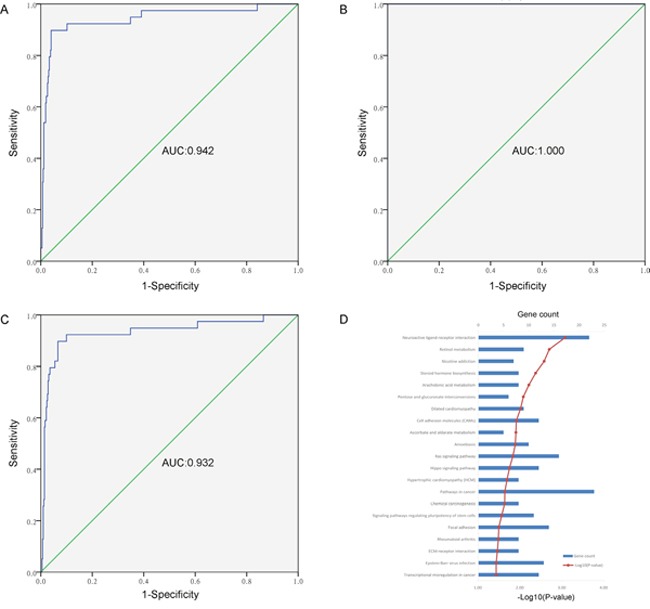
The analysis of diagnostic efficacy and gene pathway enrichment **(A)** The ROC curve of a combined diagnosis of eight CpGs (cg06363129, cg08843517, cg03576469, cg05385513, cg07220448, cg11417025 and cg20883831) in gene promoter in 423 tumor samples and 39 adjacent tissues from TCGA. **(B)** The ROC curve of a combined diagnosis of eight CpGs (cg06363129, cg08843517, cg03576469, cg05385513, cg07220448, cg11417025 and cg20883831, and cg23824801) in gene promoter in 12 tumor samples and matched adjacent tissues from GSE74013. **(C)** The ROC curve of a combined diagnosis of four CpGs (cg06363129, cg08843517, cg05385513 and cg11417025) in gene promoter in 423 tumor samples and 39 adjacent tissues from TCGA. **(D)** Gene pathway enrichment analysis for differentially hypomethylated sites between 423 tumor tissues and 39 adjacent samples in PCa.

To further explore the differentially methylated sites between tumor tissues and adjacent samples, we carried out gene pathway enrichment analysis for the matched genes. The hypermethylated sites were mainly involved in two pathways of olfactory transduction (P-value = 5.33E-10) and ribosome (P-value = 3.03E-04). The hypomethylated sites were mainly involved in 21 pathways, including neuroactive ligand-receptor interaction, cardiomyopathy, ras/hippo signaling pathway, and pathway in cancer (as shown in Figure 2D).

### Diagnostic methylation CpGs in PCa

**Figure 3:**
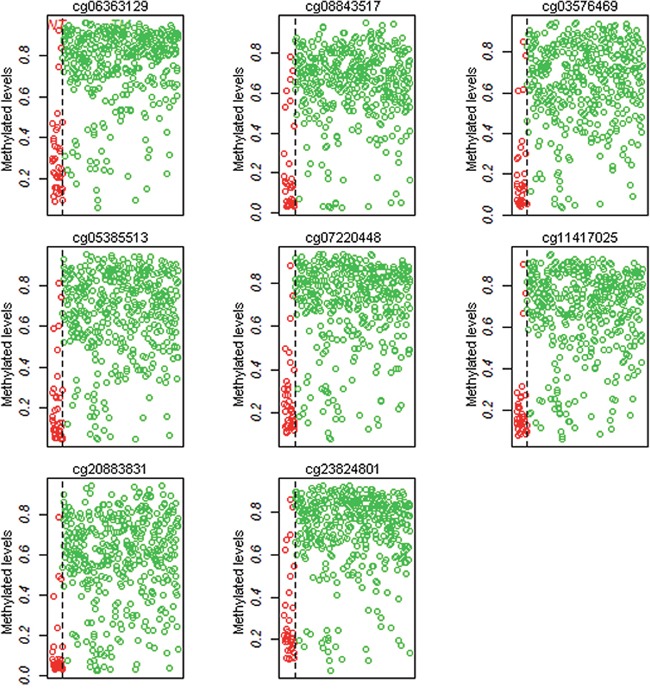
The methylation levels of eight CpGs (cg06363129, cg08843517, cg03576469, cg05385513, cg07220448, cg11417025 and cg20883831, and cg23824801) in 423 tumor samples and 39 adjacent tissues from TCGA

To investigate the diagnostic methylation markers in PCa from TCGA, we used the Prediction Analysis of Microarrays (PAM) package in Bioconductor to predict significant CpGs. After 10-fold cross-validation, we harvested eight diagnostic methylation CpGs (cg06363129, cg08843517, cg03576469, cg05385513, cg07220448, cg11417025, cg20883831, and cg23824801) in the gene promoter that were hypermethylation in PCa. Details were shown in Figure 3 and Supplementary Table 3. The Logistic regression model and the receiver operating characteristic (ROC) curves were used to evaluate the diagnostic efficiency of single site and combined diagnosis (Table 2 and Figure 2A). The area under curve (AUC) of ROC of a single site ranged from 0.910 to 0.939, and the combined diagnosis of eight sites had a higher AUC of 0.942 (95% confidence interval = 0.894-0.990, P < 0.001) than the single site. These methylation CpGs had a high diagnostic and could distinguish tumor tissues from adjacent tissues.

**Table 2:** Diagnostic efficiency of single site

Variables	AUC	Standard error	P- value	95% CI
cg06363129	0.922	0.028	0.000	0.867-0.977
cg08843517	0.913	0.027	0.000	0.860-0.966
cg03576469	0.919	0.029	0.000	0.861-0.976
cg05385513	0.927	0.024	0.000	0.879-0.975
cg07220448	0.927	0.025	0.000	0.878-0.977
cg11417025	0.923	0.029	0.000	0.867-0.979
cg20883831	0.939	0.023	0.000	0.894-0.983
cg23824801	0.910	0.028	0.000	0.855-0.965

AUC: area under roc curve; 95% CI: 95% confidence interval, P- value < 0.05 indicated a significant difference.

### Tissue specificity of diagnostic methylation CpGs in other solid tumors

To further validate the tissue specificity of eight diagnostic methylation CpGs in PCa, the datasets of other solid tumors from TCGA were analyzed. After removing the datasets that did not provide for the raw data detected by Illumina HumanMethylation450 platform, with less than 10 samples in tumors and adjacent tissues, or after neoadjuvant therapy was administered, we brought in a total of 11 solid tumors including PCa in the study (as shown in Supplementary Table 4). With P < 0.05 and |Delta Beta| > 0.2 in PCa, we found that expect for PCa, no more than two sites of these common hypermethylation of eight diagnostic methylation CpGs (cg06363129, cg08843517, cg03576469, cg05385513, cg07220448, cg11417025, cg20883831, and cg23824801) were shown in the other 10 solid tumors (bladder urothelial carcinoma, breast invasive carcinoma, colon adenocarcinoma, esophageal carcinoma, head and neck squamous cell carcinoma, kidney renal papillary cell carcinoma, liver hepatocellular carcinoma, lung adenocarcinoma, thyroid carcinoma, and uterine corpus endometrial carcinoma). A combination of theses hypermethylation CpGs had tissue specificity in PCa and may provide a novel potential biomarker for differentiating PCa from other tumors.

### Association of diagnostic methylation CpGs with Gleason Score (GS) in PCa tumors

To explore the potential roles of these eight diagnostic methylation CpGs in the biological behavior or prognosis of PCa, we assessed the methylation levels of these eight sites with different GS (Table 3). In this study, our samples included 1 cases GS6 (2 + 4), 43 cases GS6 (3 + 3), 136 cases GS7 (3 + 4), 93 cases GS7 (4 + 3), 8 cases GS8 (3 + 5), 37 cases GS8 (4 + 4), 53 cases GS8 (5 + 3), 68 cases GS9 (4 + 5), 29 cases GS9 (5 + 4), and 1 cases GS10 (5 + 5). We classified the PCa with GS into three groups (GS ≤ 6, GS = 7, and GS ≥ 8) according to the results of a previous study [5]. After using the Kruskal-Wallis test among these three groups, we found that only the cg06363129 site showed a significant difference between the groups of GS = 7 and GS ≥ 8 (P < 0.05). The high Gleason grade in PCa had increased methylation levels of cg06363129, thereby suggesting that the methylation of cg06363129 may stimulate the progression of PCa.

**Table 3:** Association of site methylation levels with Gleason score in PCa tumors

Variables	GS≤6	GS=7	GS≥8
	(n = 44)	(n = 229)	(n = 150)
cg06363129	0.825(0.753-0.886)	0.800(0.693-0.876)^a^	0.845(0.737-0.901)^a^
cg08843517	0.659(0.619-0.773)	0.681(0.561-0.767)	0.695(0.581-0.788)
cg03576469	0.674(0.549-0.790)	0.679(0.526-0.792)	0.721(0.578-0.837)
cg05385513	0.669(0.572-0.815)	0.696(0.545-0.818)	0.728(0.580-0.844)
cg07220448	0.759(0.701-0.827)	0.755(0.629-0.836)	0.802(0.663-0.857)
cg11417025	0.728(0.635-0.817)	0.705(0.559-0.810)	0.757(0.600-0.842)
cg20883831	0.631(0.461-0.764)	0.602(0.474-0.730)	0.659(0.495-0.779)
cg23824801	0.767(0.685-0.838)	0.764(0.652-0.841)	0.779(0.660-0.842)

GS: Gleason score. Data were provided as median (25%–75% quartiles). The Kruskal-Wallis test was conducted among three groups. P-value < 0.05 indicated a significant difference.

^a^: At the site of cg06363129, the groups of GS=7 and GS≥8 had a significant difference.

### Correlations of diagnostic methylation CpGs and mRNA expression

**Figure 4:**
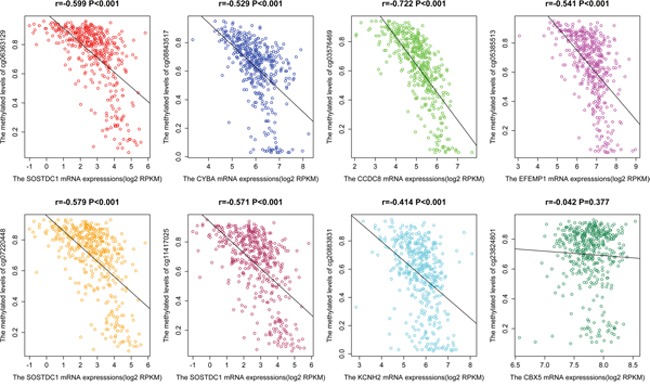
The scatter plot of the correlation of eight CpGs (cg06363129, cg08843517, cg03576469, cg05385513, cg07220448, cg11417025 and cg20883831, and cg23824801) and gene mRNA expression in PCa from TCGA

The scatter plots were carried out to describe the correlations between eight methylation CpGs and the corresponding gene mRNA expressions from TCGA (Figure 4). We observed that the correlations between cg06363129, cg07220448, or cg11417025 and SOSTDC1 expression in patients were all significant (P < 0.001). The same results were also found between cg08843517 and CYBA, cg03576469 and CCDC8, and cg05385513 and EFEMP1, in which the correlation coefficient reached 0.5, expect for cg20883831in KCNH2. However, all P-value were less than 0.001. No correlations between cg23824801 and CBX5 mRNA expression (r = - 0.042, P = 0.377) were noted.

### Verification of eight methylation sites in GEO dataset

To further verify the methylation levels of sites in PCa, the GSE74013 dataset was analyzed in the study. The diagnostic sites (cg06363129, cg08843517, cg03576469, cg05385513, cg07220448, cg11417025, cg20883831, and cg23824801) showed significantly higher methylation in PCa than that in the adjacent tissues (P < 0.01) (Supplementary Figure 1). At the same time, an AUC of 1.000 (95%CI = 1.000-1.000, P < 0.001) was found for the combined diagnosis of eight sites on GEO dataset (Figure 2B). These results supported the data from TCGA.

### Detection of methylation sites through pyrosequencing

**Figure 5:**
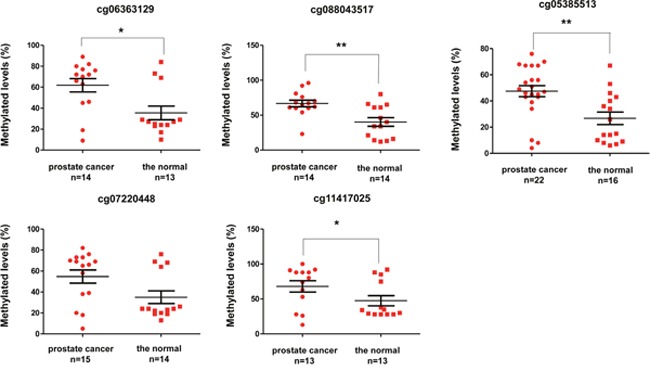
Methylation levels between prostate cancer and normal tissues through pyrosequencing

To further improve the application value of the combined diagnosis, we excluded the lowest tissue-specific site of cg03576469 that was hypermethylated in several solid cancers, such as breast invasive carcinoma, colon adenocarcinoma, esophageal carcinoma, and lung adenocarcinoma. Additionally, cg20883831 in KCNH2 and cg23824801 in CBX5 were also excluded due to low correlation coefficient (r < 0.5). At last, we verified five methylated sites (cg06363129, cg07220448, cg11417025 in SOSTDC1; cg08843517 in CYBA; and cg05385513 in EFEMP1) in PCa and normal tissues, including adjacent tissues and benign prostatic hyperplasia (BPH) through pyrosequencing (Figure 5). The methylation levels of cg06363129, cg08843517, cg05385513, and cg11417025 were higher in PCa than that in the normal tissues (P < 0.05). Compared with normal tissues, the methylation levels of cg07220448 had no statistical differences in PCa (P = 0.0631). These findings further confirmed the results of TCGA and GEO from experimental data.

Based on the results from TCGA, GEO and our experiment, we found that the combined diagnosis of four methylation sites (cg06363129, cg08843517, cg05385513, and cg11417025) had tissue specificity in PCa than other solid tumors (Supplementary Table 4), and a high diagnostic efficacy that reached up to 0.932 in TCGA (Figure 2C), 1.000 in GEO, and 0.899 in our samples, thereby indicating that these markers could be better used in clinical research.

## DISCUSSION

DNA methylation occurring in the 5’-CG-3’ gene sequence is considered as two nucleotides cytosine DNA methylation of CG being selectively added to form 5-methylcytosine. In tumorigenesis, the methylation model tends to be highly modified on CpG islands, resulting in reduction and inactivation of gene expression. In our study, we analyzed the differentially methylated sites of 423 primary PCas and 39 adjacent samples from TCGA database and found that DNA hypomethylation mainly occurs in PCa, and differentially methylated sites are mainly located on CpG islands and TSS200 region in the gene promoter. Additionally, eight methylation sites (cg06363129, cg07220448, and cg11417025 in SOSTDC1; cg08843517 in CYBA; cg03576469 in CCDC8; cg05385513 in EFEMP1; cg20883831in KCNH2; and cg23824801in CBX5) with high tissue specificity and diagnostic efficacy were mined from TCGA, and the dataset from GEO further proved this result. Among them, the methylation levels of five CpG sites (cg06363129, cg08843517, cg03576469, cg07220448, and cg11417025) had negative correlations with gene expressions (r > 0.5 and P < 0.001) and the high methylation of cg06363129 may stimulate the progression of PCa. Pyrosequencing in our samples confirmed that the combined diagnosis of four methylation sites (cg06363129, cg08843517, cg05385513, and cg11417025) had high diagnostic efficacy in PCa and could be better used in clinical research.

Sclerostin domain containing protein 1(SOSTDC1) is a member of the sclerostin family. SOSTDC1was reported to be involved in the Wnt/bone morphogenetic protein signaling in bone metabolism [14]. In addition, it also suppresses tumorigenesis. Results of TCGA and GEO dataset showed that the diagnostic methylation CpGs (cg06363129, cg07220448, and cg11417025) in PCa located at promoter region of SOSTDC1 and the methylation levels of sites had negative correlations with SOSTDC1 mRNA expression. SOSTDC1 regulates the expressions of p21Cip and p27Kip to inhibit the proliferation of non-small-cell lung cancer cells [15]. SOSTDC1 inhibits cell proliferation and differentiation and induces G1/S arrest in thyroid cancer [16]. In prostate cells, researchers have found that SOSTDC1 regulates the levels of hepcidin by inhibiting BMP and Wnt signaling, and the promoter methylation of SOSTDC1 is associated with tumor recurrence in PCa [17]. These results suggest that the high methylation model at cg06363129 or cg11417025 may decrease SOSTDC1 expression, promoting PCa development. Gleason Score, which is associated with the biological behavior and prognosis, has been recognized as an important reference index in PCa. In our study, high Gleason grade in PCa had greater methylation levels of cg06363129, suggesting that high methylation of cg06363129 may stimulate the progression of PCa.

Similar results were also found for cg08843517 located in cytochrome b light chain (CYBA). CYBA, which is encoded by a polymorphic gene, is involved in the process of electron transport and superoxide anion production [18]. CYBA has been reported to be associated with melanoma through DNA hypermethylation during tumor progression [19]. CYBA also displays increased methylation in several invasive cell lines in malignant melanoma [20]. In prostate cancer cells, androgens can induce oxidative stress and radiation resistance by increasing the expressions of p22^phox^ and gp91^phox^ [21]. CYBA is also involved in the biological processes of angiogenesis and tumor growth by regulating AKT and ERK1/2 signaling pathways [22]. In our study, the high methylation of cg08843517 is related to CYBA mRNA expression, and this may provide a novel idea for exploring the mechanism of PCa.

The EGF-containing fibulin-like extracellular matrix protein 1 (EFEMP1), an extracellular matrix (ECM) protein, is associated with the tumorigenesis in different types of carcinoma [23]. In our study, the diagnostic methylation of cg05385513, which is located at the promoter region of EFEMP1, had higher methylation level in tumor than that in the normal tissues. From the tissue level, high methylation in EFEMP1 promoter may down-regulate mRNA expression, leading to cell proliferation [24]. From the cell level, by studying PCa cell lines, EFEMP1 mRNA expression was confirmed to be significantly lower in PCa than in the normal tissues [25]. The cg05385513 may play an important role in the promoter region of EFEMP1 that regulates cell adhesion and proliferation [25].

In conclusion, we utilized bioinformatics method to mine the diagnosis methylation CpGs from TCGA, and we found that the combined diagnosis of four methylation CpGs (cg06363129, cg08843517, cg05385513, and cg11417025) have high tissue specificity and diagnosis efficacy in PCa. The cg06363129 located in SOSTDC1 may be involved in the progression of PCa. These results revealed a novel potential biomarker for diagnosis of the PCa.

## MATERIALS AND METHODS

### Methylation analysis of TCGA data

All data (clinical parameters, methylation levels, and gene expressions) of PCa and adjacent tissues were obtained from TCGA up to October 10, 2015. The adjacent tissues were defined as a distance that was greater than 2cm from tumor margin. All patients with a history of other malignancies or those who received neoadjuvant therapy (discrepancy) or radiation therapy were excluded in this study. The methylation values, which came from Illumina HumanMethylation450 BeadChips, were obtained from TCGA using RnBeads software (version 0.99) as previous [26]. The batch effects and surrogate variables were removed with the Surrogate Variable Analysis (SVA) package in R (http://www.bioconductor.org) [27]. The diagnostic methylation markers in PCa were explored using the PAM package in Bioconductor, and the gene enrichment analysis was carried out on David Bioinformatics Resources (version 6.8, NIAID/NIH) (https://david-d.ncifcrf.gov). To further validate the diagnostic markers in PCa, the diagnostic efficacy of the methylation CpGs was evaluated using the ROC curves, and the relationships between the tissue specificity the prognosis (PCa with GS), gene expression (level 3 data, RNA-seq Version 2), and the methylation levels of CpGs were analyzed.

### Patient samples

A total of 13 paired PCa and adjacent tissues were collected through surgical resection in Hunan Province Tumor Hospital, and nine PCa and five BPH biopsy samples were obtained from the 303rd Hospital of Chinese People's Liberation Army, Guangxi, China from June 15, 2015 to April 30, 2016 with informed consent obtained from all individual participants (Table 4). All patients had primary PCa and without history of chemotherapy or radiotherapy. After surgical resection or biopsy, the fresh specimens were determined by a pathologist as previously described [5] and immediately stored at -80°C until analysis. This study was approved by the Medical Ethics Committee of Guangxi Medical University.

**Table 4:** Basic characteristics of prostate cancer samples used in pyrosequencing analysis

Variables	Prostate cancer (n=22)	The normal (n=18)
**Age(years)**	68.00(65.50,72.25)	64.00(67.00,71.25)
**Category**		
Adjacent	—	13
BPH	—	5
**Tumor size**		
T1	0	0
T2	8	8
T3	5	5
T4	0	0
NA	9	5
**Lymph node**		
N0	11	11
N1	1	1
NA	10	6
**Metastasis status**		
M0	12	12
M1	0	0
NA	10	6
**Gleason Score**		
GS ≤ 6	4	1
GS =7	9	6
GS ≥ 8	8	6
NA	1	5

NA: not available. The ages were presented as the median (25%–75% quartiles).

### Validation of GEO dataset

The methylation data of PCa on Illumina HumanMethylation450 array were searched on GEO database (http://www.ncbi.nlm.nih.gov/geo/) by using keywords “(prostate carcinoma or prostate cancer) and (methylation)” up to June 12, 2016. The inclusion criteria for datasets in the study were (1) the datasets provided the raw data of PCa and adjacent tissues, (2) sample source was restricted to humans, (3) the sample was not involved in adjuvant therapy, and (4) the number of PCa and adjacent samples was more than 10. Finally, only one dataset (GSE74013) was included in our study, and this contained 12 PCa and 12 matched adjacent samples. The methylation levels were calculated using β-value as previously described: β = methylated intensity/(methylated intensity + unmethylated + 100) [11].

### Quantitative pyrosequencing

DNA was extracted with Genomic DNA Extraction Kit (TaKaRa, Dalian, China). A total of 500 ng DNA was bisulfite converted through EZ DNA Methylation-Gold Kit^TM^ (D5005, Zymo Research, United States). We tested five methylation sites for validation through pyrosequencing. The specific primers were designed with Pyromark Assay Design software (version 2.0; Qiagen) (the primers were shown in Supplementary Table 1), and the methylation sites were included in the analysis sequence. Three different CpG island loci (cg06363129, cg07220448, cg11417025) were close to each other that located at the genomic position of 16505602, 16505592, and 16505589 in SOSTDC1 respectively in Supplementary Table 3. Therefore, they had the same primer that can measure the methylated levels of three loci simultaneously. The polymerase chain reaction (PCR) was carried out by using PyroMark PCR Kits (Qiagen). A total of 25 μl volume was amplified with 50 ng bisulfite-converted DNA, 1×Pyromark PCR master Mix 12.5 μl, 1×CoralLoad Concentration 2.5 μl, 8ul RNase-free water, 0.5ul 0.2Um forward, and 0.5 μl 0.2 Um 5’biotinylated-reverse primer. Thermal cycling conditions included an initial denaturation at 95 °C for 15 min, followed by 45 cycles of denaturation (30 s at 94 °C), annealing (30 s at 56 °C), and extension (30 s at 72 °C), and then final extension at 72 °C for 10 min and cooling to 4 °C until analysis. Pyrosequencing was carried out to detect the methylation levels of CpG sites through PyroMark Q96 ID Software (Qiagen) according to the manufacturer's instruction.

### Statistical analysis

The methylation levels of CpGs were presented as the median (25%–75% quartiles). The differences in the tumor and the normal tissues were provided as Delta Beta. The Mann-Whitney U or the Wilcoxon's matched pairs test was conducted between two groups, and the Kruskal-Wallis test was used among three groups. The associations between methylation sites and gene mRNA expression were assessed through Pearson's rank-correlation coefficients. The logistic regression model and an AUC of ROC curves with 95% CI were used to predict diagnostic efficacy of the combination of methylation sites as previously described [28]. Statistical analysis was performed with IBM SPSS (Version 20.0, Inc., Chicago, IL, USA) and GraphPad Prism (Version 5.0, GraphPad Software; San Diego, CA, USA). The statistical tests were two-tailed, and *P* < 0.05 was regarded as statistically significant.

## SUPPLEMENTARY MATERIALS FIGURE AND TABLES








