# Supplementary material for: Promoter DNA methylation analysis reveals a combined diagnosis of CpG-based biomarker for prostate cancer
Source: Oncotarget. 2017 Mar 22;8(35):58199–209. doi: 10.18632/oncotarget.16437 (PMC5601644; doi:10.18632/oncotarget.16437)
Supplement: Supplementary file 1 [file oncotarget-08-58199-s001.pdf]

Promoter DNA methylation analysis reveals a combined diagnosis of CpG-based biomarker for prostate cancer

SUPPLEMENTARY FIGURE AND TABLES

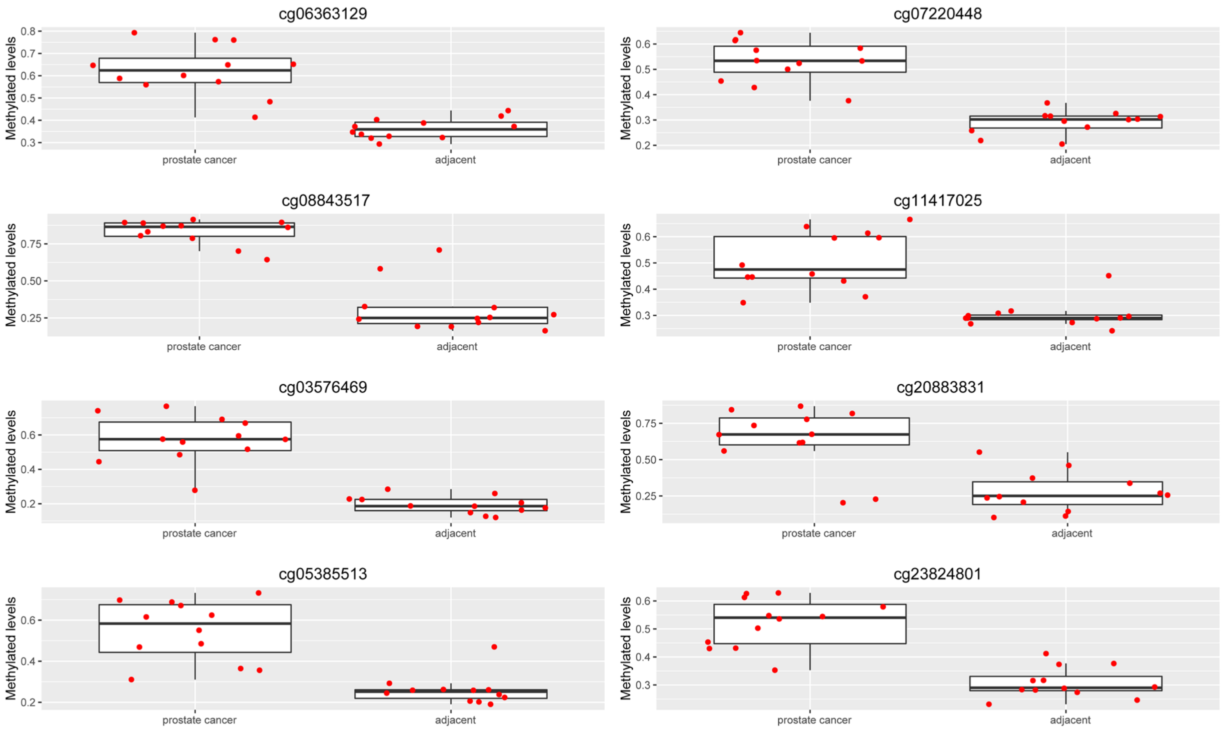

Supplementary Figure 1: The methylation levels of eight CpGs (cg06363129, cg08843517, cg03576469, cg05385513, cg07220448, cg11417025, cg20883831, and cg23824801) in 12 tumor samples and matched adjacent tissues from GSE74013.

Supplementary Table 1: The specific primers of methylated sites

Sites	Gene	Primer	5'- 3' base sequence
		F	GTAGAAAAGGAGGAGTTTGGTATAT
cg06363129	SOSTDC1	R	CAAAACAACTATAAAAAAACCAATCTCTT
		S	ATATAAATTGTTTGAAATTTTAGAG
		F	GTAGAAAAGGAGGAGTTTGGTATAT
cg07220448	SOSTDC1	R	CAAAACAACTATAAAAAAACCAATCTCTT
		S	ATATAAATTGTTTGAAATTTTAGAG
		F	GTAGAAAAGGAGGAGTTTGGTATAT
cg11417025	SOSTDC1	R	CAAAACAACTATAAAAAAACCAATCTCTT
		S	ATATAAATTGTTTGAAATTTTAGAG
		F	GGTTTGGTTAGGTAGGGAGATGA
cg05385513	EFEMP1	R	ACCAACCCACAAAACTTTACCCATAA
		S	TAGGGAGATGAGGTT
		F	GGGATATTTTTTTAGGTTGTAGTTTTTAT
cg08843517	CYBA	R	ACAACCCTACACCCTACAAATA
		S	GATAAGAATTAGGTTGGGATATTG

F: forward primer; R: reverse primer; S: sequencing primer.

Supplementary Table 2: 1585 differential CpGs within promoter

See Supplementary File 1

Supplementary Table 3: Diagnostic methylation CpGs in prostate cancer

See Supplementary File 2

Supplementary Table 4: The diagnostic methylation CpGs in 11 kinds of solid tumors

See Supplementary File 3
