# Supplementary material for: Promoter DNA methylation analysis reveals a combined diagnosis of CpG-based biomarker for prostate cancer
Source: Oncotarget. 2017 Mar 22;8(35):58199–209. doi: 10.18632/oncotarget.16437 (PMC5601644; doi:10.18632/oncotarget.16437)
Supplement: Supplementary file 4 [file oncotarget-08-58199-s004.docx]

**Supplementary Table 4 The diagnostic methylation CpGs in 11 kinds of solid tumors**

| **Type** | **Samples** | **cg06363129** | | **cg08843517** | | **cg03576469** | | **cg05385513** | | **cg07220448** | | **cg11417025** | | **cg20883831** | | **cg23824801** | |
| --- | --- | --- | --- | --- | --- | --- | --- | --- | --- | --- | --- | --- | --- | --- | --- | --- | --- |
|  |  | **Delta Beta** | **P-value** | **Delta Beta** | **P-value** | **Delta Beta** | **P-value** | **Delta Beta** | **P-value** | **Delta Beta** | **P-value** | **Delta Beta** | **P-value** | **Delta Beta** | **P-value** | **Delta Beta** | **P-value** |
| BLCA | 299T+19N | 0.103 | < 0.001 | 0.622 | < 0.001 | 0.190 | < 0.001 | 0.004 | 0.807 | 0.078 | 0.003 | 0.095 | 0.001 | 0.061 | < 0.001 | 0.164 | < 0.001 |
| BRCA | 719T+93N | 0.210 | < 0.001 | 0.118 | < 0.001 | 0.364 | < 0.001 | 0.269 | < 0.001 | 0.198 | < 0.001 | 0.234 | < 0.001 | 0.110 | < 0.001 | 0.181 | < 0.001 |
| COAD | 277T+11N | -0.035 | 0.181 | 0.070 | < 0.001 | 0.245 | < 0.001 | 0.287 | < 0.001 | -0.004 | 0.839 | 0.001 | 0.943 | -0.031 | 0.185 | -0.053 | 0.043 |
| ESCA | 162T+10N | 0.087 | 0.097 | 0.021 | 0.496 | 0.301 | < 0.001 | 0.241 | < 0.001 | 0.075 | 0.204 | 0.075 | 0.214 | 0.135 | < 0.001 | 0.241 | < 0.001 |
| HNSC | 484T+14N | 0.120 | < 0.001 | 0.046 | 0.094 | 0.159 | < 0.001 | 0.053 | < 0.001 | 0.106 | 0.001 | 0.114 | < 0.001 | 0.045 | 0.026 | 0.275 | < 0.001 |
| KIRP | 202T+34N | -0.034 | 0.042 | 0.025 | 0.250 | 0.109 | < 0.001 | 0.066 | < 0.001 | -0.025 | 0.094 | -0.013 | 0.311 | 0.316 | < 0.001 | 0.034 | 0.065 |
| LIHC | 327T+46N | -0.090 | < 0.001 | -0.073 | 0.001 | 0.137 | < 0.001 | 0.121 | < 0.001 | -0.065 | < 0.001 | 0.012 | 0.456 | 0.087 | < 0.001 | 0.129 | < 0.001 |
| LUAD | 386T+17N | 0.135 | < 0.001 | 0.091 | 0.006 | 0.284 | < 0.001 | 0.045 | 0.005 | 0.172 | < 0.001 | 0.201 | < 0.001 | 0.191 | < 0.001 | 0.122 | < 0.001 |
| THCA | 456T+20N | 0.105 | 0.001 | -0.005 | 0.356 | 0.051 | 0.087 | -0.013 | 0.353 | 0.060 | 0.034 | 0.053 | 0.050 | -0.017 | 0.582 | -0.013 | 0.554 |
| UCEC | 390T+25N | -0.080 | 0.001 | 0.025 | 0.211 | 0.097 | < 0.001 | 0.146 | < 0.001 | -0.064 | 0.005 | -0.052 | 0.012 | 0.102 | < 0.001 | 0.146 | < 0.001 |
| PRAD | 423T+39N | 0.462 | < 0.001 | 0.462 | < 0.001 | 0.469 | < 0.001 | 0.467 | < 0.001 | 0.449 | < 0.001 | 0.468 | < 0.001 | 0.476 | < 0.001 | 0.432 | < 0.001 |

T: tumor; N: normal; BLCA: bladder urothelial carcinoma; BRCA: breast invasive carcinoma; COAD: colon adenocarcinoma; ESCA: esophageal carcinoma; HNSC: head and

neck squamous cell carcinoma; KIRP: kidney renal papillary cell carcinoma; LIHC: liver hepatocellular carcinoma; LUAD: lung adenocarcinoma; THCA: thyroid carcinoma; UCEC:

uterine corpus endometrial carcinoma; PRAD: prostate adenocarcinoma. Data were provided as Delta Beta. The Student’s *t* -test was conducted between the tumor and the normal. P-value < 0.05

indicated a significant difference.
